# SBE6: a novel long-range enhancer involved in driving sonic hedgehog expression in neural progenitor cells

**DOI:** 10.1098/rsob.160197

**Published:** 2016-11-16

**Authors:** Nezha S. Benabdallah, Philippe Gautier, Betul Hekimoglu-Balkan, Laura A. Lettice, Shipra Bhatia, Wendy A. Bickmore

**Affiliations:** 1 MRC Human Genetics Unit, Institute of Genetics and Molecular Medicine, University of Edinburgh, Crewe Road, Edinburgh EH4 2XU, UK; 2 Edinburgh Super Resolution Imaging Consortium (ESRIC), Institute of Genetics and Molecular Medicine, University of Edinburgh, Crewe Road, Edinburgh EH4 2XU, UK

**Keywords:** sonic hedgehog, long-range enhancer, neural progenitor cells

## Abstract

The expression of genes with key roles in development is under very tight spatial and temporal control, mediated by enhancers. A classic example of this is the sonic hedgehog gene (*Shh*), which plays a pivotal role in the proliferation, differentiation and survival of neural progenitor cells both *in vivo* and *in vitro. Shh* expression in the brain is tightly controlled by several known enhancers that have been identified through genetic, genomic and functional assays. Using chromatin profiling during the differentiation of embryonic stem cells to neural progenitor cells, here we report the identification of a novel long-range enhancer for Shh—Shh-brain-enhancer-6 (SBE6)—that is located 100 kb upstream of *Shh* and that is required for the proper induction of *Shh* expression during this differentiation programme. This element is capable of driving expression in the vertebrate brain. Our study illustrates how a chromatin-focused approach, coupled to *in vivo* testing, can be used to identify new cell-type specific *cis*-regulatory elements, and points to yet further complexity in the control of *Shh* expression during embryonic brain development.

## 1. Introduction

**Figure 1.**
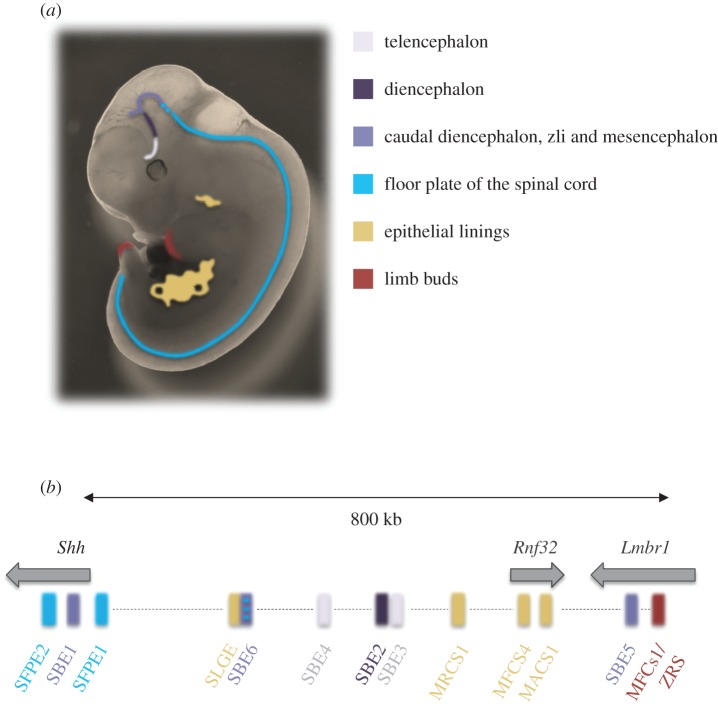
The sonic hedgehog (*Shh*) regulatory region. (*a*) Cartoon shows the sites of *Shh* enhancer activity in the E11.5 mouse embryo. Sites of *Shh* expression in the forebrain (telencephalon, diencephalon), caudal diencephalon, zli and midbrain/mesencephalon, floor plate, epithelial linings of gut and lung, and the distal limb bud are indicated with different colours. (*b*) Genomic map of the *Shh* regulatory region on mouse chromosome 5 indicating the known tissue-specific *Shh* enhancers, colour-coded as in (*a*).

Enhancers orchestrate the regulation of gene expression, which is critical for cell lineage specification and differentiation, and they therefore have a pivotal role during embryonic development [1]. A well-defined example of such *cis*-regulatory control is seen in the case of the sonic hedgehog (*Shh*) gene. *Shh* encodes a secreted signalling protein that imparts patterns of growth and identity to cells during many stages of embryonic development, including neural progenitors throughout ventral regions of the developing central nervous system (CNS) [2–4] (figure 1*a*).

*Shh* is located at one end of a large (approx. 1 Mb) regulatory domain containing a number of known enhancers controlling various *Shh* expression domains [5–12] (figure 1*b*). Precise *Shh* expression is critical for proper spinal cord and brain development, and this is governed by a subset of floor-plate and brain enhancers, many of which were identified by reporter assays. Shh floor-plate enhancer SFPE1, located 8 kb upstream of the *Shh* transcription start site (TSS), drives expression in the ventral spinal cord, and SFPE2 and Shh-brain-enhancer 1 (SBE1), positioned in the second intron of *Shh*, show activity in the floor plate of the spinal cord, as well as the ventral midbrain (mesencephalon), ventroposterior region of the diencephalon and the zona limitans intrathalamica (zli) [5,6]. An enhancer trap assay—using BAC transgenes to screen the *Shh* regulatory region—identified SBE2, SBE3 and SBE4 that drive *Shh* expression in the diencephalon (SBE2) and in the telencephalon (SBE4) [7]. Most recently, a combined informatics and experimental study identified SBE5 that drives expression in the zli [13].

Perturbation of Shh *cis*-regulation leads to severe neural defects in mammals. Translocations separating SBE2, 3 and/or 4 from *Shh,* disrupt the function of these enhancers and lead to *Shh* haploinsufficiency, causing diverse holoprosecencephaly (HPE) phenotypes [14,15], point mutation that results in the loss of SBE2 activity in the hypothalamus also leads to HPE [16]. Together, these observations highlight the importance of reporting and understanding new *cis*-regulatory elements that control *Shh* expression in the CNS.

Using chromatin profiling during the *in vitro* differentiation of mouse embryonic stem cells (mESCs) to neural progenitor cells (NPCs), we report a new Shh brain enhancer (SBE6) that we show is necessary for proper *Shh* expression in NPCs and that is active in vertebrate brain and neural tube development in transgenic assays.

## 2. Material and methods

### 2.1. Cell culture and neural differentiation

46c mouse embryonic stem cells (mESCs), derived from E14tg2A, contain a GFP insertion into the *Sox1* locus [17]. mESCs were cultured and differentiated into NPCs for 5 or 7 days with N2B27 medium as described previously [18]. To sort GFP^+^ cells after transfection or differentiation, cells were trypsinized and resuspended in PBS + 10% medium. Flow cytometric analysis was performed, using the 488 nm laser of a BD FACSAriaII SORP (Becton Dickinson) with 525/50 nm bandpass filters. BD FACSDiva software (Becton Dickinson, v. 6.1.2) was used for instrument control and data analysis.

### 2.2. Quantitative analysis of gene expression

RNA was prepared from approximately 1 × 10^6^ 46c mESCs or NPCs, using the RNeasy mini kit (Qiagen) according to the manufacturer's protocol, including a DNaseI (Qiagen) treatment for 15 min at room temperature. cDNA was synthesized from 2 µg purified RNA with superscript II reverse transcriptase (Invitrogen) primed with random hexamers (Promega). Real-time (q)PCR was carried out on a Roche LightCycler 480 real-time PCR system, using a LightCycler 480 Sybr Green detection kit (Roche) as described previously [19]. Primer pairs for qRT-PCR are listed in electronic supplementary material, table S1.

The real-time thermal cycler was programmed as follows: 15 min Hotstart; 44 PCR cycles (95°C for 15 s, 55°C for 30 s, 72°C for 30 s). The relative mRNA expression for each primer set in each sample was measured by the LightCycler software and normalized to the mean for *Gapdh* from at least two biological replicates and technical triplicates.

### 2.3. Native chromatin immunoprecipitation and microarray analysis

Nuclei from 3 × 10^6^ mESCs or sorted Sox1+ NPCs were prepared and resuspended in NB-R (85 mM NaCl, 5.5% sucrose, 10 mM Tris–HCl pH 7.5, 3 mM MgCl_2_, 1.5 mM CaCl_2_, 0.2 mM PMSF, 1 mM DTT) as previously described [20]. Micrococcal nuclease (MNase) digestion and native chromatin immunoprecipitation (ChIP) were performed as previously described [21,22]. Antibodies used for ChIP were H3K4me1 (Abcam ab8895) and H3K27ac (Millipore 07-360).

Ten nanograms (optimal) of input or ChIP DNA were amplified, using the WGA2 whole genome amplification kit (Sigma). Amplified material was labelled with Cy3 or Cy5 by random priming according to the NimbleGen ChIP-chip protocol (Roche). In total, two or three biological replicates with dye swaps were hybridized for 20 h and washed according to the manufacturer's protocol. A custom 3 × 720 K mouse tiling array (NimbleGen, Roche) containing 179 493 unique probes from different genomic regions was used, with each probe represented by four replicates. Arrays were scanned on a NimbleGen MS 200 Microarray scanner (Roche), using 100% laser power and 2 µm resolution. Raw signal intensities were quantified from TIFF images, using MS 200 Data Collection software.

Microarray data were analysed in R, using the bioconductor packages Beadarray and Limma according to the Epigenesys NimbleGen ChIP-on-chip protocol 43 (www.epigenesys.eu/en/protocols/bioinformatics). Scale normalization was used within replicates, to control interarray variability. Each condition was represented by two biological replicates hybridized as dye swap experiments and enrichment scores are defined as log_2_ ChIP/input signal.

### 2.4. Computational analysis of the SBE6 region

Evolutionary conservation of the SBE6 region was assessed, using the ‘Vertebrate Multiz Alignment & Conservation/Multiz Alignments and Conserved Elements’ tracks in the UCSC genome browser [23]. This delineated the following subregions for further analysis:

SBE6.1: Chr5: 28 889 688–28 890 461, SBE6.2: Chr5: 28 893 935–28 895 000 (mm9)

rVista [24] was used to align the mouse and human orthologous sequences, with the default sequence aligner (LAGAN) and default parameters. Transcription factor binding sites (TFBS) for known forebrain transcription factors [25] available on the rVista server were selected (Arx, Maf, Dlx5, Pbx1, ER81, Six3, Vax1).

JASPAR [26] was used independently on the mouse and human core sequences, searching for potential neural activity present in the Jaspar Core Vertebrata matrices list (DLX6, PBX1, ETV1, Six3, SP8 and VAX1) with the default parameters (relative profile score threshold 80%; electronic supplementary material, table S2). Hits were then highlighted on the rVista alignment.

### 2.5. Zebrafish enhancer reporter assay

The putative SBE6.1 and SBE6.2 enhancers were cloned by PCR amplification of the relevant fragment and flanking sequence from mouse genomic DNA, using Phusion high fidelity polymerase (NEB) and the following primers:

Sbe6.1 Fw B4 : **AGGGGAGAACTTTGTATAGAAAAGTTGGCGCGC**CCACCTGCTTCTCTGAGGAA
Sbe6.1 Rv B1R : **AGGGGACTGCTTTTTTGTACAAACTTG**CTTAGGCCATTGTGCCCAC
Sbe6.2 Fw B4 : **AGGGGAGAACTTTGTATAGAAAAGTTGGCGCGC**TGAAGTCAAGGGCCTGGTACT
Sbe6.2 Rv B1R : **AGGGGACTGCTTTTTTGTACAAACTTG**ATCAGCCCTCCAGTTTGACT
Negative controls used were sequences 3′ of *Shh*, which have no suspected regulatory activity, and which are the same genomic distance from *Shh* as SBE6.1 and SBE6.2 are upstream (5′) of *Shh*.
Negative controls:
Sbe6.1 Fw B4: **AGGGGAGAACTTTGTATAGAAAAGTTGGCGCGC**CGAGTGCAGGTGTTTGTGAA
Sbe6.1 Rv B1R: **AGGGGACTGCTTTTTTGTACAAACTTG**CCTCAACACAGCATTGCCAA
Sbe6.2 Fw B4: **AGGGGAGAACTTTGTATAGAAAAGTTGGCGCGC**AGAGAGTGAAGATTCCCAGCT
Sbe6.2 Rv B1R: **AGGGGACTGCTTTTTTGTACAAACTTG**TGAGGCAGTGTCTATCTTTTGAC

attB4 and attB1r sequences (bold) were included in the PCR primers for use with the Gateway recombination cloning system (Invitrogen, 12538120). The amplified fragment was first cloned into the Gateway pP4P1r entry vector and sequenced using M13 forward and reverse primers for verification. The elements in the pP4P1r vector were combined with a pDONR221 construct containing either a Gata2 promoter-eGFP- polyA or a Gata2 promoter mCherry-polyA cassette [27], and recombined into a destination vector with a Gateway R4-R2 cassette flanked by Tol2 recombination sites.

Reporter plasmids were isolated using Qiagen miniprep columns and were further purified using a Qiagen PCR purification column (Qiagen), and diluted to 50 ng µl^−1^ with DNAse/RNAse free water. Tol2 transposase RNA was synthesized from a *Not*I-linearized pCS2-TP plasmid using the SP6 mMessage mMachine kit (Ambion), and similarly diluted to 50 ng µl^−1^. Equal volumes of the reporter construct(s) and the transposase RNA were mixed immediately prior to injections. 1–2 nl of the solution was microinjected per zebrafish embryo at the one- to two-cell stage for up to 200 embryos. Embryos were screened for fluorescence at 1–5 days post-fertilization (i.e. 24–120 hours post-fertilization, hpf) and raised to adulthood. Germline transmission was identified by mating of sexually mature adults to wild-type fish and examining their progeny for fluorescence. F_1_ embryos from three to five F_0_ lines showing the best representative expression pattern for each construct were selected for confocal imaging. A few positive embryos were also raised to adulthood, and F_1_ lines were maintained by outcrossing. A summary of the independent lines analysed for each construct and their expression sites is included in electronic supplementary material, table S3. Imaging of zebrafish reporter transgenic embryos was carried out as previously described [27].

### 2.6. Mouse transgenic reporter assays

The same SBE6.1 PCR amplicon, with attB4 and attB1r sequences included as used for reporter assays in zebrafish (above), were used to generate enhancer-reporter constructs for mouse transgene assays. The amplicon was cloned directly into an hsp68-LacZ vector containing a P4-P1r entry cassette [28]. Transgenic mice were generated by microinjection into mouse oocytes, and the analysis of transgenic lines was carried out as previously described [28]. Two independently derived E11.5 SBE6.1-LacZ embryos were independently analysed; one a transient insertion, the second from a stable line. For analysis, embryos were dissected in PBS and left in LacZ fix for 1 h (1% formaldehyde; 0.2% glutaraldehyde; 2 mM MgCl_2_; 5 mM EGTA; 0.02% NP-40 in PBS). After fixation the embryos were washed in PBS containing 0.02% NP-40, before being stained overnight at 37°C in the dark in a solution containing 5 mM K_3_Fe(CN)_6_; 5 mM K_4_Fe(CN)_6_.3H_2_O; 2 mM MgCl_2_; 0.01% sodium deoxycholate; 0.02% NP-40 and 0.1% 5-bromo-4-chloro-3-indolyl-β-d-galactopyranoside (X-gal). Embryos were then fixed with 4% PFA and photographed on a Leica MZ FLIII Microscope fitted with a Hamamatsu Orca-ER digital camera and a CRI microcolour filter.

### 2.7. mRNA *in situ* hybridization

RNA *in situ* hybridization on fish embryos was performed as previously described [29]. The sequences of primers used for synthesis of *Shh* hybridization probes are the following:

Forward primer (5′-SP6 promoter-sequence-3′) AAGCTGACACCTCTCGCCTA and reverse primer (5′-T7 promoter-sequence-3′) GAGCAATGAATGTGGGCTTT.

Mouse *in situ* hybridization was performed with DIG-labelled gene-specific antisense probes as previously described [30]. The Shh probe was provided by McMahon [31].

### 2.8. Deletion of SBE6 from the 46c embryonic stem cell genome

Cell line deletions were produced, using the Crispr/cas9 system. SBE6.1- and SBE6.2-specific gRNA primers (electronic supplementary material, table S4) were cloned into the cas9 plasmid pX458 following protocols from the Zhang laboratory [32–34]. 46C mESCs were transfected with the resulting plasmids using Lipofectamine 2000 reagent (Invitrogen cat. no. 11668) following the manufacturer's recommendations as described in [19]. Single transfected cells were sorted based on GFP expression from pX458 and cultured further. DNA extraction and genotyping were performed 7 days after sorting, using overnight incubation at 55°C with lysis buffer (10 mM TrisHCl pH 7.5, 10 mM EDTA, 10 mM NaCl, 0.5% SDS, 1 mg ml^−1^ ProteinaseK) followed by ethanol precipitation and washes. Genomic DNA was amplified with the following primers:

SBE6.1 Fw: TTTTGGAAGCTTAAATGCCCAT
SBE6.1 Rv: CCACCACAAGCACATTCAT
SBE6.2 Fw: GCCTCCATGAAGTCCAATGG
SBE6.2 Rv: CCACCCTTGCTACTCAGGAA

Amplification was done using DreamTaq Green PCR master mix (ThermoFisher K1081) following the manufacturer's protocol and PCR products were assessed by agarose gel electrophoresis. Amplified products were later sequenced to further confirm homozygous deletions.

## 3. Results

### 3.1. SBE6.1 and SBE6.2, two new putative *cis*-regulatory elements active in neural progenitor cells

**Figure 2.**
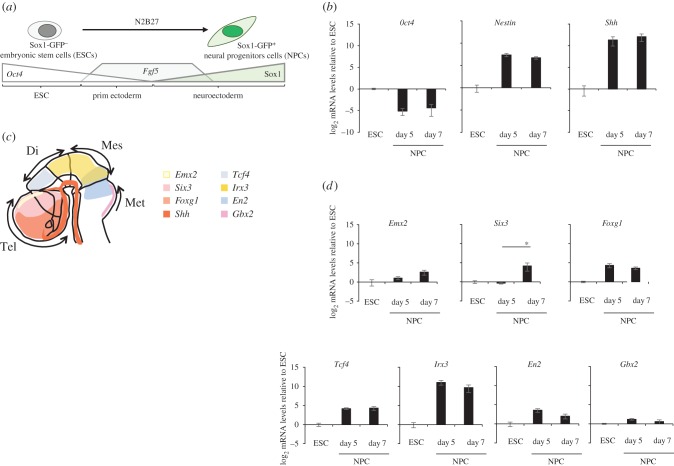
Sox1-GFP+ neural differentiation from ESC to NPCs. (*a*) Schematic shows the differentiation of 46c mouse embryonic stem cells (ESC)—which are Sox1-GFP^–^ and express high levels of the pluripotency factor *Oct4*—into first primitive ectoderm as *Oct4* levels decrease and fibroblast growth factor 5 (*Fgf5*) levels rise, and then further into neuroectoderm as *Fgf5* levels start to decrease and Sox1 levels rise, allows for the purification of Sox1-GFP^+^ neural progenitor cells (NPC). (*b*) qRT-PCR showing mean ± s.e. of the mean (s.e.m.) log_2_ mRNA levels for *Oct4*, *Nestin* and *Shh* in ESCs and in NPCs after 5 and 7 days of differentiation. Expression levels are relative to *Gapdh* and normalized to ESC mRNA levels. Data are from three biological replicates and technical triplicates. (*c*) Schematic of an E11.5 mouse brain and gene expression markers patterning the telencephalon (tel), diencephalon (di), mesencephalon (mes) and metencephalon (met). (*d*) qRT-PCR shows means (±s.e.m.) of log_2_ mRNA levels of marker genes for different brain regions in ESC and NPC differentiated for 5 or 7 days. As in (*b*), levels are relative to *Gapdh* and normalized to ESC mRNA levels. *Six3* mRNA levels significantly increase in NPCs between days 5 and 7 of differentiation (one-tailed Student's *t*-test; *p* = 0.023). Data consist of five biological replicates and technical triplicates.

We used the differentiation of 46c mESCs as a model system to identify putative regulatory elements that may become activated concomitant with the expression of *Shh* during neural differentiation. These cells contain a knockin of GFP into the *Sox1* locus allowing for the monitoring of neural differentiation and the purification, by fluorescence-activated cell sorting (FACS), of Sox1+ neuroepithelial progenitor cells (NPCs; figure 2*a*) [17,35,36]. Sox1+ cells appear after day 3 of differentiation, and from day 3 to 7, expression of *Shh* and *Nestin* increase while *Oct4* mRNA levels progressively decrease (figure 2*b*). Analysis of these NPC cells for expression of markers from different regions of the developing brain (figure 2*c*) suggests that these cells do not have a distinct regional identity, though there is some evidence for a slight shift towards a more telencephalic fate (increasing *Six3* and *Emx2* expression) and away from the hindbrain (decreasing *En2* and *Gbx2* expression) by day 7 (figure 2*d*).

**Figure 3.**
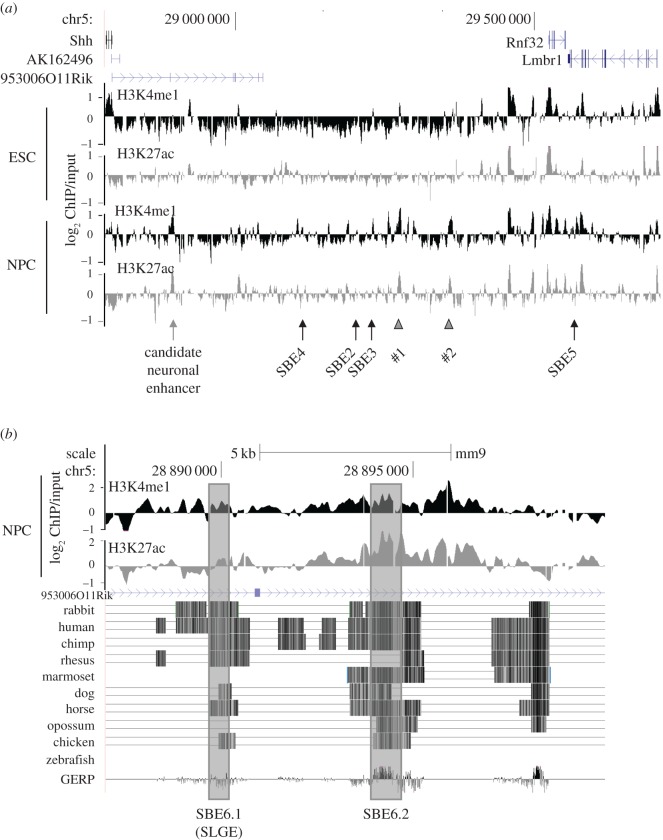
Chromatin immunoprecipitation analysis during NPC differentiation. (*a*) Log_2_ native ChIP/input MNase-digested chromatin for H3K4me1 (black) and H3K27ac (grey) from ESCs and Sox1^+^ NPCs purified after 5 days of differentiation. Averages of data from two biological replicates are shown. The position of genes (above) and known neural enhancers for *Shh* (below) are shown; grey arrow indicates the new candidate neural enhancer SBE6; grey arrowheads indicate two other regions that gain active enhancer signatures in NPCs. Genome coordinates are from the mm9 assembly of the mouse genome (chr5: 28 782 000–29 711 000 bp). (*b*) Zoom-in of the region (chr5: 28 887 000–28 900 000) of putative NPC enhancer activity shows conservation across multiple vertebrate species. Two smaller core conserved regions named SBE6.1 (=SLGE) and SBE6.2 are indicated.

Genome-wide ChIP has allowed the identification of several post-translational histone modification characteristics of active enhancers including H3K4me1 and H3K27ac [37]. The use of these two histone marks is widely employed to identify new active enhancer elements in the genome [38], though they are not comprehensive [18,39]. Using native ChIP coupled to hybridization on microarrays (ChIP-chip) that tile the whole *Shh* regulatory region, we assessed the sites of enriched H3K4me1 and H3K27ac in mESCs (where *Shh* is not expressed) and in Sox1+ NPC after 5 days of neural differentiation. Significant gains of H3K4me1 and H3K27ac were not detected at the known SBE2, 3, 4 or 5 brain enhancers (figure 3*a*). However, a prominent change in the ChIP profile was seen at a small region approximately 100 kb upstream of the *Shh* TSS. This region has no evidence of active enhancer marks in mESCs but gains both H3K4me1 and H3K27ac upon neural differentiation (figure 3*a*).

Analysis of sequence conservation across multiple vertebrate species indicated that this region contains two blocks of evolutionary conservation in mammals and birds, and we named these putative NPC enhancers SBE6.1 (mm9 coordinates Chr5: 28 889 688–28 890 461, 96 048 bp upstream of *Shh* TSS) and SBE6.2 (Chr5: 28 893 935–28 895 000, 100 295 bp away from *Shh*; figure 3*b*). Interestingly, two other sequences beyond SBE3 also show a gain of active enhancer marks (arrowheads in figure 3*a*), but are not investigated further here.

*In silico* motif analysis using UCSC comparative genomics of SBE6.1 and SBE6.2 allowed us to identify two core (approx. 1 kb) regions that are highly conserved. Complementary rVista, JASPAR and RSAT scans of those regions revealed the presence of predicted binding sites for neural transcription factors such as ETV1, SP8, VAX1 and DLX6 (electronic supplementary material, table S2).

The SBE6.1 sequence is entirely included in a recently described 1.7 kb lung and gut epithelium regulatory element for *Shh* expression in mouse embryos called SLGE (chr5: 28 889 230–28 890 979) [40], raising the possibility either that this enhancer has multiple regulatory activities or that SLGE is ectopically activated in NPCs. SBE6.2 has not previously been identified or studied.

### 3.2. SBE6.1 drives expression in the brain of developing zebrafish and mouse embryos

**Figure 4.**
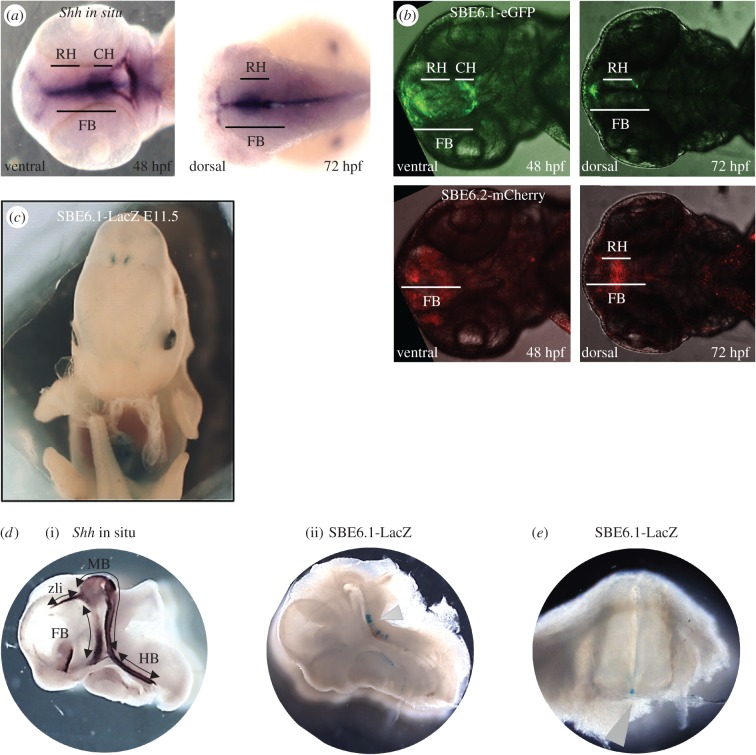
Enhancer reporter assays for SBE6.1 and SBE6.2. (*a*) *Shh* mRNA *in situ* hybridization on zebrafish embryos. A ventral view is shown at 48 h post-fertilization (hpf) and a dorsal view at 72 hpf. *Shh* expression is detected in the rostral hypothalamus (RH) and caudal hypothalamus (CH) of the forebrain (FB). (*b*) Confocal microscopy of 48 and 72 hpf zebrafish embryos from stable transgenic lines carrying a Tol2 transposon with SBE6.1 and SBE6.2, driving GFP and mCherry, respectively. Reporter gene expression is detected in the rostral hypothalamus (RH) and caudal hypothalamus (CH) of the forebrain (FB). (*c*) External view of the LacZ staining in a stable SBE6.1 transgenic E11.5 embryos shows expression in a portion of diencephalon cells. (*d*) (i) *Shh* mRNA *in situ* hybridization in an E11.5 mouse embryo displaying expression in the forebrain (telencephalon, diencephalon; FB), midbrain (caudal diencephalon, zona limitans intrathalamica (zli) and mesencephalon; MB), and hindbrain (HB). (ii) sagittal section of an E11.5 transient SBE6.1-LacZ transgenic embryo with arrowhead indicating staining in a portion of the ventral mesencephalon, with some cells expressing SBE6.1 near the hindbrain. (*e*) E11.5 transient SBE6.1-LacZ transgenic embryo with arrowhead indicating staining in the floor plate of the spinal cord.

To test the regulatory potential of SBE6.1 and SBE6.2, we used a zebrafish Tol2 transposon assay in which the test element is juxtaposed to a minimal promoter driving the expression of either GFP or mCherry reporter gene expression. This assay has been shown to recapitulate the correct expression pattern for the SBE2 enhancer and to detect the loss of this enhancer activity associated with mutation of a SIX3 binding site found in the cases of HPE [16,27]. *In situ* hybridization for *Shh* mRNA in wild-type zebrafish embryos reveals expression in the forebrain at 48 and 72 hpf [27]. Using this assay, SBE6.1 enhancer activity was detected in the developing forebrain of the zebrafish embryos in four independent stable transgenic lines from 30 to 72 hpf (figure 4*a*,*b*; electronic supplementary material, table S3). SBE6.2 however failed to consistently drive reporter gene expression in the forebrain, with forebrain-specific activity noted in only one out of the four independent transgenic lines generated (electronic supplementary material, table S3). Therefore, SBE6.1 has a consistent enhancer function and is active in zebrafish forebrain development.

The ability of SLGE to drive expression in the developing mouse brain is unclear, but it is known to be capable of driving expression in the brain of transgenic rabbits [40]. We therefore made mouse transgenics to analyse the regulatory potential of SBE6.1 in mouse development. LacZ staining of transient and stable SBE6.1 transgenic embryos revealed activity in the pharyngeal endoderm, gut and cloaca of the mouse embryo as expected owing to the overlap with SLGE (electronic supplementary material, figure S1*a*,*b*). X-gal staining could be also detected in few superficial diencephalon cells where *Shh* is not expressed (figure 4*c*; electronic supplementary material, figure S1*c*). However, SBE6.1 also showed activity in the developing ventral mesencephalon with some cells expressing SBE6-LacZ near the hindbrain and as well as in the ventral midline of the mouse embryonic neural tube—all sites of endogenous *Shh* expression (figure 4*d*,*e*; electronic supplementary material, figure S1*d*).

SBE6.1 is only active in a small number of cells in transgenic embryos, and we cannot at this stage confirm how accurately this recapitulates a subset of endogeneous *Shh* expression. However, the strong similarities between the two mouse embryos do support our conclusion that the SBE6.1 enhancer is capable of activity in the developing vertebrate brain, from a forebrain pattern in zebrafish transgenics to a floor plate and ventral mesencephalon expression in mouse transgenic embryos.

### 3.3. SBE6.1 enhances *Shh* expression in neural progenitor cells

**Figure 5.**
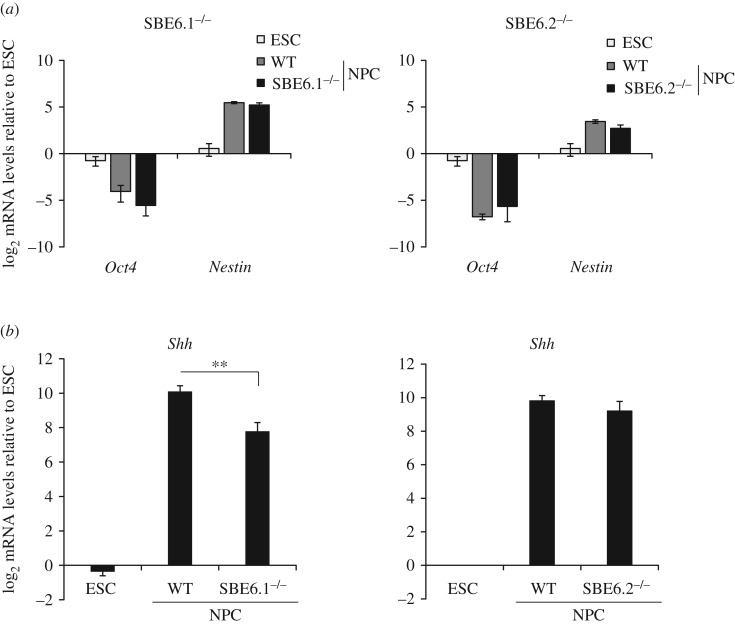
Shh expression levels in neural precursor cells (NPC) derived from ESC lines with SBE6.1 or SBE6.2 deletions. (*a*) qRT-PCR shows mean (±s.e.m.) log_2_ mRNA levels of *Oct4* and *Nestin* in wild-type 46c ESC, and NPCs, and in NPCs derived from SBE6.1^−/−^ (left) or SBE6.2^−/−^ (right) 46c cells. Levels are relative to *Gapdh* and normalized to levels in wild-type ESCs. (*b*) As in (*a*) qRT-PCR shows mean (± s.e.m.) log_2_ *Shh* mRNA levels in wild-type NPCs, and in NPCs derived from 46c cell lines deleted for SBE6.1 (left) or SBE6.2 (right). mRNA levels are shown relative to *Gapdh* and normalized to those in wild-type ESCs. *Shh* mRNA levels are significantly reduced in NPCs derived from SBE6.1^−/−^ cell lines after 7 days of differentiation (one-tailed Student's *t*-test; *p*-value = 0.002). ESC data consist of three biological replicates, SBE6.1^−/−^ dataset are six biological replicates from two independent deletion cell lines compared with six biological replicates of wild-type (WT) NPC. SBE6.2^−/−^ data are from three biological replicates from three independent deletion cell lines with three biological replicates of wild-type (WT) NPCs.

To determine the regulatory activity of SBE6.1 and SBE6.2 in their native context, we used CRISPR/Cas9 to delete these elements from the genome in 46c mESCs (SBE6.1^−/−^ and SBE6.2^−/−^; electronic supplementary material, figure S2). We generated and analysed two SBE6.1^−/−^ and three SBE6.2^−/−^ independent cell lines. Upon NPC differentiation, the proportion of Sox1-GFP^+^ cells remained the same between NPCs derived from wild-type and SBE6.1^−/−^ or SBE6.2^−/−^ cells, analysis of *Oct4* and *Nestin* mRNA expression confirmed that differentiation of mESCs into NPC was not perturbed by the loss of either SBE6.1 or SBE6.2 (figure 5*a*). However, in NPCs derived from SBE6.1^−/−^ but not SBE6.2^−/−^ cells, levels of *Shh* expression were significantly reduced compared with wild-type cells (one-tailed Student's *t*-test; *p* = 0.002). Average *Shh* mRNA levels in NPCs differentiated from SBE6.2^−/−^ ESCs were not significantly different relative to wild-type (figure 5*b*).

Together, these data suggest that SBE6.1 is a long-range enhancer that contributes to driving *Shh* expression during the differentiation of ESCs to neural progenitor cells.

## 4. Discussion

The regulation of *Shh* is a paradigm for the complex control of gene expression at different times and places in development. More than 10 discrete enhancers have been identified in the large (approx. 1 Mb) *Shh* regulatory domain [12] (figure 1*b*). Most of these enhancers were identified using transgenic reporter assays [7]. Others have been identified through genetics in mouse and man when mutations in *Shh* enhancers cause phenotypes that result from aberrant control of specific aspects of *Shh* expression in development. Most recently, information on transcription factor motifs in known *Shh* brain enhancers has been used to search for other similar patterns of motifs in the *Shh* regulatory domain and has identified a new enhancer that drives *Shh* expression in a discrete region of the brain [7,13].

Here, we show that analysis of histone modifications (H3K4me1 and H3K27ac), typically associated with active enhancers, in an *in vitro* neural differentiation system can be used to identify a new enhancer that is important for the activation of *Shh* in neural progenitor cells. This enhancer, which we have named SBE6, is located 100 kb 5′ of *Shh* and is activated during the differentiation of mESCs to Sox1+ NPCs. Analysis of transcription factor motifs suggests that SBE6 contains consensus binding sites for a number of transcription factors expressed in the brain. Using an enhancer reporter assay in zebrafish and mouse, we show that *in vivo* the SBE6.1 region of SBE6, but not SBE6.2, can drive expression in the developing brain. Consistent with this, genetic ablation of SBE6.1 in mESCs, but not SBE6.2, abrogates the induction of *Shh* expression during *in vitro* NPC differentiation. Therefore, despite the presence of strong active enhancer histone modifications in NPCs, we find no functional evidence that SBE6.2 is a neural enhancer, highlighting that precise annotation and understanding of regulatory regions of the genome requires confirmation via functional enhancer assays.

**Figure 6.**
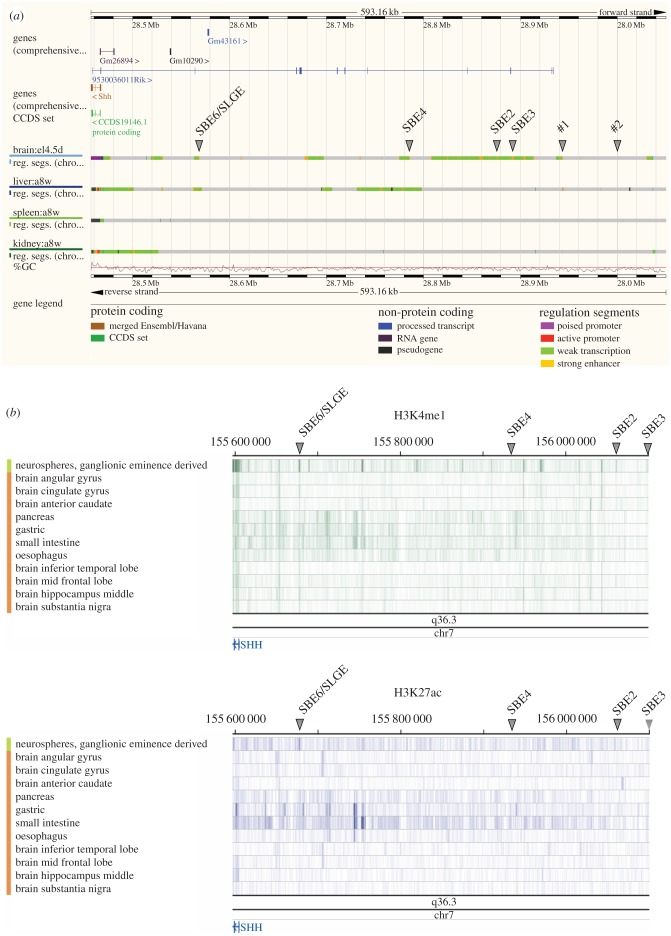
Chromatin state discovery and characterization (ChromHMM) in mouse and human Shh regulatory region. (*a*) Ensembl *Mus musculus* v. 84.38 (GRCm38.p4) view of chr5: 28 456 840–29 050 000 with regulatory feature tracks form primary cells (embryonic E14.5 brain, and adult liver, spleen and kidney) from ChromHMM. Grey arrowheads indicate previously described enhancers and two putative sequences (#1 and #2) indicated in figure 3. The position of SLGE/SBE6 is indicated. (*b*) Roadmap epigenome browser view of the human Shh locus on Hg19 (chr7: 155 595 558–156 100 554), showing H3K4me1 (top) and H3K27ac (bottom tracks) ChIP-seq from a variety of brain regions, including neurospheres, as well as pancreas, gastric, small intestine, oesophagus, tissues. Arrowheads indicate the corresponding positions of SLGE/SBE6, SBE4, SBE2 and SBE3.

Our analyses presented here add to the growing number of functionally validated enhancers directing *Shh* expression in different developmental contexts. Given the large size of the gene desert upstream of *Shh*, where many of these enhancers are located, there is the potential for this region to harbour many more *cis*-regulatory elements and, given the complexity of brain development, many of these may be enhancers active in the brain. Indeed, regulatory segmentation built from ChromHMM or Segway using ENCODE data from various mouse primary tissues indicates the presence of several regions with chromatin signatures indicative of enhancer activity in mouse brain at E14.5—a period of mouse development when neurogenesis is ongoing (figure 6*a*). This includes the genomic regions containing the known neural enhancers SBE2–4, but also a region that corresponds to SBE6. The many other regions called as likely active enhancers using the analysis from just four tissues (brain, liver, spleen and kidney) at one embryonic stage (figure 6*a*) suggests that the *Shh* regulatory region may harbour many tens of as yet unannotated enhancers. We note that the new sites detected by these high-throughput methods (marked with arrowheads as #1 and #2 in figure 6*a*) correspond to peaks of H3K4me1/H3K27ac that are induced during the differentiation of 46c mESCs to NPCs (figures 3 and 6*a*; electronic supplementary material, figure S3). A similar analysis of chromatin profiling data from the Roadmap project also indicates the signature of an active neural enhancer at the position of SBE6 in material from different regions of the human brain and particularly in ganglionic eminence derived neurospheres (figure 6*b*). This analysis also indicates many other potential regulatory elements active in different brain regions.

It is interesting that the genome coordinates of SBE6.1 are completely contained within those reported for the *Shh* lung and gut epithelium regulatory element SLGE [40]. Transgenic analysis in the rabbit had shown that the mouse SLGE fragment can drive expression in the rabbit brain [40]. Here, we have shown that SBE6.1 can drive expression in the brain of zebrafish and mouse. Although we cannot completely exclude that our observations of SBE6.1 transgenic reporter expression in the vertebrate brain and neural tube represents ectopic activity of SLGE in these assays, our chromatin profiling indicates that this region does harbour active regulatory potential in Sox1+ NPCs. Consistent with this, ENCODE and Roadmap data also indicate that this region of the mammalian genome has active enhancer chromatin marks in neural tissue, as well as in the liver (figure 6*a*; electronic supplementary material, figure S3) and gastric tissue (figure 6*b*). Important sequences required for enhancer function work as assemblies of transcription factor motifs [13]. SBE6.1 and SLGE motifs may be intermingled but still specific to a precise tissue and stage of development, or may be overlapping to various extents. There are several other examples of regulatory elements capable of driving expression at multiple sites during development—for example, the global control region 5′ of HoxD contains regulatory information capable of driving expression in the CNS and in the limb [41]. Moreover, for *SOX9* and *PAX6*, there are *cis*-regulatory elements driving expression in multiple developmental sites, and in which disease-associated variants have been identified that ablate enhancer function in one tissue but leave the other sites of expression unaltered [27,42]. Further analysis will be necessary to determine the critical transcription factor binding sites in SBE6.1/SLGE needed to drive enhancer function in different developmental settings.

## Supplementary Material

Supplementary data list

## Supplementary Material

Supplementary Figure 1

## Supplementary Material

Supplementary Figure 2

## Supplementary Material

Supplementary Figure 3

## Supplementary Material

Supplementary Table 1

## Supplementary Material

Supplementary Table 2

## Supplementary Material

Supplementary Table 3

## Supplementary Material

Supplementary Table 4

## Data accessibility

H3K4me1 and H3K27ac ChIP-on-chip data is deposited in the GEO repository (http://www.ncbi.nlm.nih.gov/geo/; accession no. GSE84168).
