# Supplementary material for: SBE6: a novel long-range enhancer involved in driving sonic hedgehog expression in neural progenitor cells
Source: Open Biol. 2016 Nov 16;6(11):160197. doi: 10.1098/rsob.160197 (PMC5133441; doi:10.1098/rsob.160197)
Supplement: Supplementary data list [file rsob160197supp1.docx]

# SBE6, a novel long-range enhancer involved in driving Sonic Hedgehog expression in neural progenitor cells.

Nezha S. Benabdallah^1,2^, Philippe Gautier^1^, Betul Hekimoglu-Balkan^1^, Laura A. Lettice^1^, Shipra Bhatia^1^, Wendy A. Bickmore*^1^

**Supplementary data**

### **Supplementary Figure 1**. Mouse SBE6.1 Transgenic reporter.

### **Supplementary Figure 2**. Genomic PCR of SBE6.1^-/-^ and SBE6.2 ^-/-^ ESC lines.

### **Supplementary Figure 3.** Chromatin state discovery and characterization (ChromHMM) in SBE6.1, SBE6.2 and peak #2.

**Supplementary Table 1.** Primer pairs used for qRT-PCR analys is of mRNA expression

**Supplementary Table 2.** Jaspar scores for forebrain transcription factor binding sites in mouse

and human SBE6.1/SBE6.2.

**Supplementary Table 3.** Cis-Regulatory Element (CRE) driven transgene expression sites in F1

zebrafish embryos

**Supplementary Table 4.** Primers used for gRNAs for CRISPR/Cas9 targeting of SBE6.1 and

SBE6.2
