# Supplementary material for: SBE6: a novel long-range enhancer involved in driving sonic hedgehog expression in neural progenitor cells
Source: Open Biol. 2016 Nov 16;6(11):160197. doi: 10.1098/rsob.160197 (PMC5133441; doi:10.1098/rsob.160197)
Supplement: Supplementary Figure 1 [file rsob160197supp2.pdf]

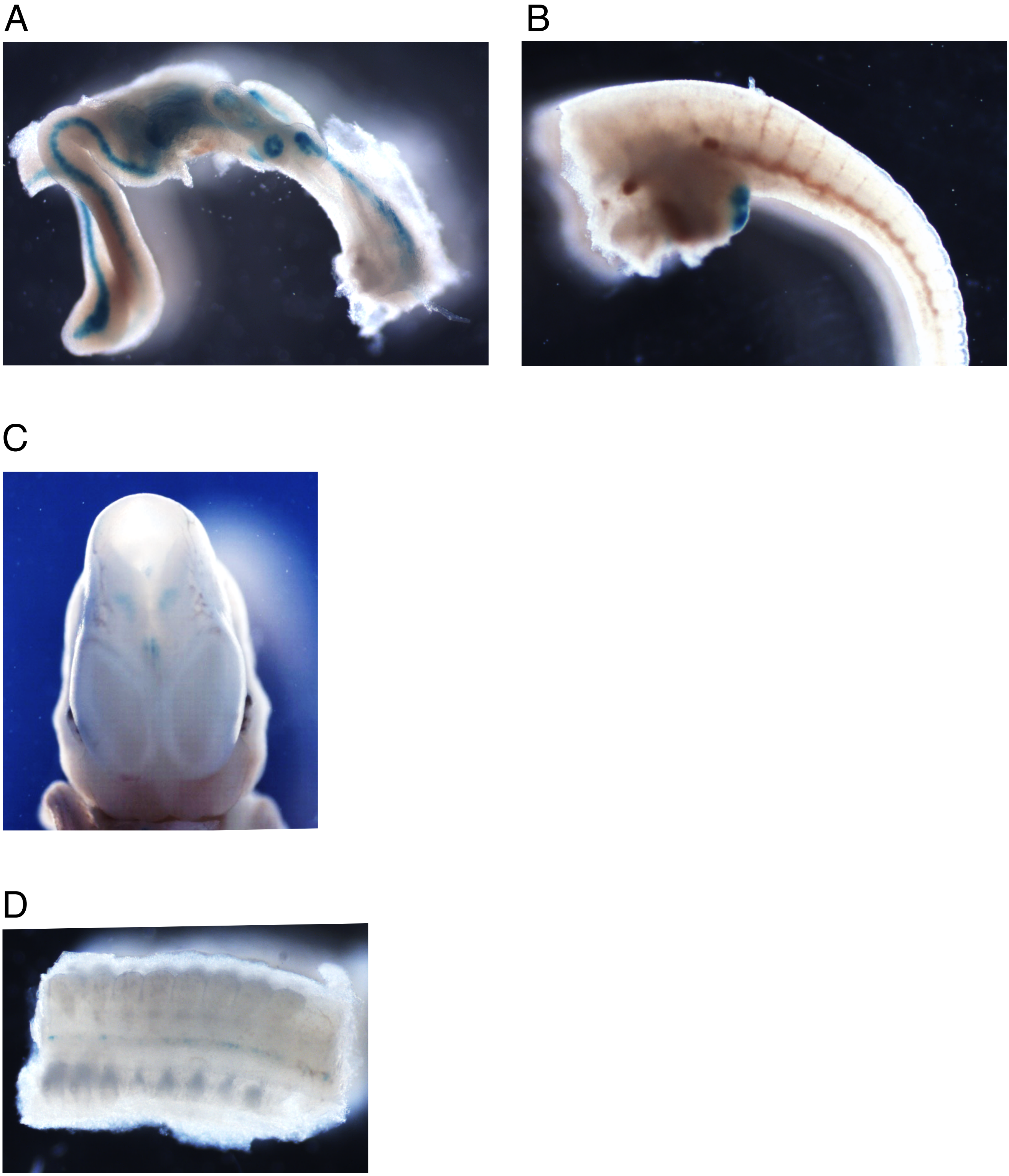

Supplementary Figure 1. Mouse SBE6.1 Transgenic reporter.
A) LacZ staining in the gut of a transient SBE6.1-lacZ mouse embryo at E11.5. B) LacZ staining in the cloaca (right) of a transient SBE6.1-lacZ mouse embryo at E11.5. C) LacZ staining in the brain of a transient SBE6.1-lacZ mouse embryo at E11.5. D) LacZ staining in the floor-plate of the spinal cord in a transient SBE6.1 transgenic embryo.
