# Supplementary material for: SBE6: a novel long-range enhancer involved in driving sonic hedgehog expression in neural progenitor cells
Source: Open Biol. 2016 Nov 16;6(11):160197. doi: 10.1098/rsob.160197 (PMC5133441; doi:10.1098/rsob.160197)
Supplement: Supplementary Figure 2 [file rsob160197supp3.pdf]

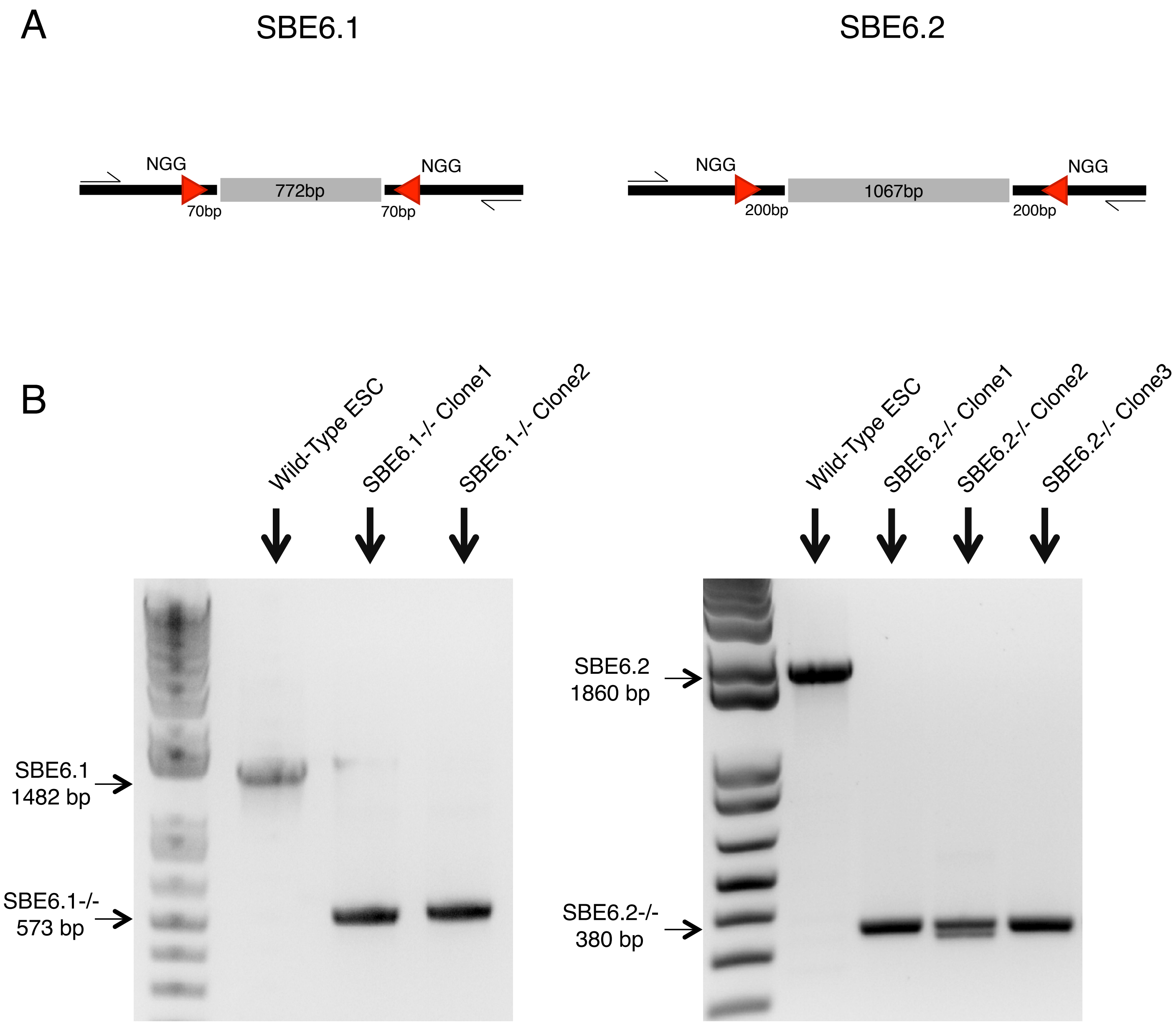

Supplementary Figure 2. Genomic PCR of SBE6.1-/- and SBE6.2 -/- ESC lines.
A) Illustration showing SBE6.1 and SBE6.2 sequences with the position of the gRNAs (arrowheads) used for CRISPR/Cas9 mediated targeted deletions. Half arrows indicate the position of PRC primers used for genotyping. B) Agarose gels showing PCR amplification of (left) SBE6.1 in wild-type 46C mESCs, SBE6.1-/- ESC clones 1 and 2, and (right) SBE6.2 in wild-type cells and in SBE6.2-/- ESC clones 1, 2 and 3,
