# Supplementary material for: SBE6: a novel long-range enhancer involved in driving sonic hedgehog expression in neural progenitor cells
Source: Open Biol. 2016 Nov 16;6(11):160197. doi: 10.1098/rsob.160197 (PMC5133441; doi:10.1098/rsob.160197)
Supplement: Supplementary Figure 3 [file rsob160197supp4.pdf]

A

SBE6.1

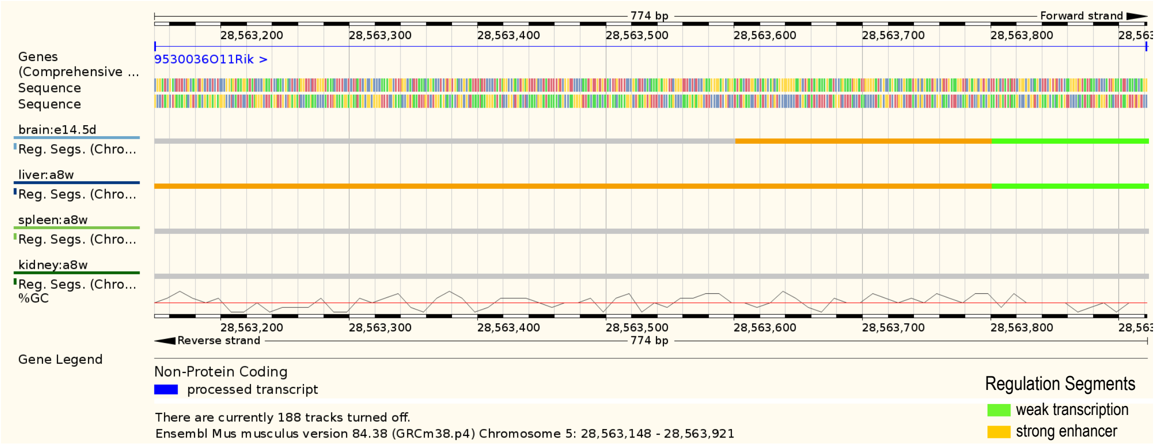

B

SBE6.2

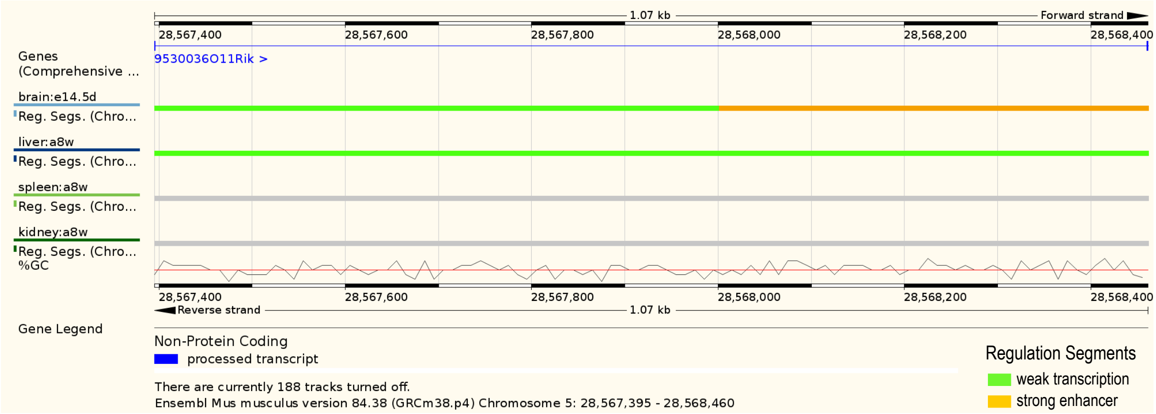

C

#2

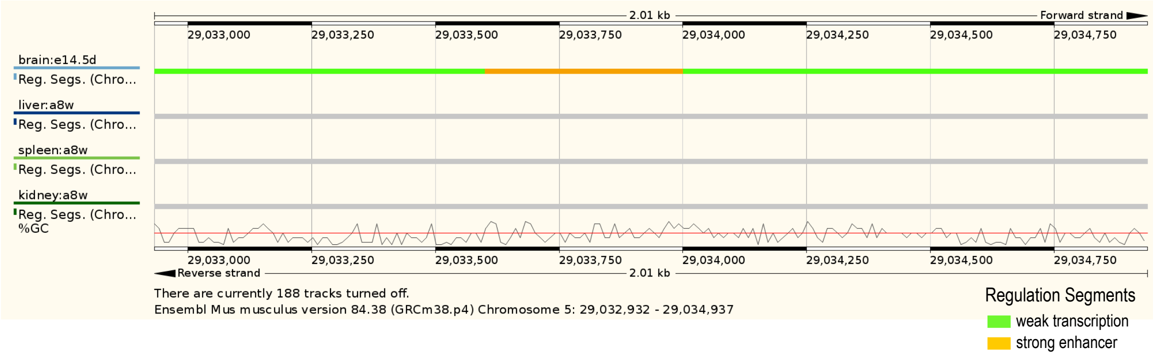

Supplementary Figure 3. Chromatin state discovery and characterization (ChromHMM) in SBE6.1, SBE6.2 and peak #2.
Ensembl Mus musculus genome version 84.38 (GRCm38.p4) view of regulatory feature tracks from ChromHMM for the regions corresponding to (A) SBE6.1, (B) SBE6.2 and (C) putative peak #2 from Figure 3)
