# Supplementary material for: SBE6: a novel long-range enhancer involved in driving sonic hedgehog expression in neural progenitor cells
Source: Open Biol. 2016 Nov 16;6(11):160197. doi: 10.1098/rsob.160197 (PMC5133441; doi:10.1098/rsob.160197)
Supplement: Supplementary Table 1 [file rsob160197supp5.docx]

### Supplementary Table 1. Primer pairs used for qRT-PCR analysis of mRNA expression

| Gapdh Fw | ATCACCATCTTCCAGGAGCGAG | | | |
| --- | --- | --- | --- | --- |
| Gapdh Rv | GACCCTTTTGGCTCCACCCTTC | | | |
| Oct4 (Pou5f1) Fw | CGAGAACAATGAGAACCTTC | | | |
| Oct4 (Pou5f1) Rv | CCTTCTCTAGCCCAAGCTGAT | | | |
| Nestin Fw | GATCGCTCAGATCCTGGAAG | | | |
| Nestin Rv | AGGTGTCTGCAAGCGAGAGT | | | |
| Shh Fw | ACGATTTAAGGAACTCACCC | | | |
| Shh Rv | TTGTCTTTGCACCTCTGAG | | |  |
|  |  |  |  |  |
| Foxg1 Fw | GCTGGACATGGGAGATAGGA | | | |
| Foxg1 Rv | GGTGGTGATGATGATGGTGA | | | |
| Six3 Fw | CCCACACAAGTAGGCAACTGGT | | | |
| Six3 Rv | CTGAGCCGTGCGTGGGGCAG | | | |
| Emx2 Fw | GCTCATCCACCGCTACCGATAT | | | |
| Emx2 Rv | GCTCTAGCCTTAAAAGCTGGGAC | | | |
| Tcf4 Fw | CCTCCAATCCTTCAACTCCTGTG | | | |
| Tcf4 Rv | TCCAAACGGTCTTCGATTCGGC | | | |
| Irx3 Fw | AGCCGCAGGTCATCCCGCTG | | | |
| Irx3 Rv | CGGCTGGAAAGCTGTCTTGAGT | | | |
| En2 Fw | GGTCTACTGCACGCGCTATTCT | | | |
| En2 Rv | AAACTCAGCCTTGAGCCTCTGG | | | |
| Gbx2 Fw | ATGCGGAAGACGGCAAAGCCTT | | | |
| Gbx2 Rv | CCACCTTTGACTCGTCTTTCCC | | | |
|  |  | | | |
|  |  | | | |
|  |  |  |  |  |
|  |  | | | |
|  |  | | | |
|  |  | | | |
|  |  | | | |
|  |  | | | |
|  |  | | | |
|  |  | | | |
|  |  | | | |
|  |  |  |  |  |
|  |  | | | |
|  |  | | | |
