# Supplementary material for: SBE6: a novel long-range enhancer involved in driving sonic hedgehog expression in neural progenitor cells
Source: Open Biol. 2016 Nov 16;6(11):160197. doi: 10.1098/rsob.160197 (PMC5133441; doi:10.1098/rsob.160197)
Supplement: Supplementary Table 2 [file rsob160197supp6.docx]

### Supplementary Table 2. Jaspar scores for forebrain transcription factor binding sites in mouse and human SBE6.1/SBE6.2.

**Predicted putative sites in mouse SBE6.1**

| Model ID | Model name | Score | Relative score | predicted site sequence |
| --- | --- | --- | --- | --- |
| MA0070.1 | PBX1 | 9.456 | 0.82183775 | AGATCACTCAAG |
| MA0747.1 | SP8 | 8.565 | 0.84123614 | CACAACCCCACT |
| MA0747.1 | SP8 | 8.342 | 0.83709184 | CCCTCCCCCACC |
| MA0747.1 | SP8 | 6.623 | 0.80514545 | AGCACTCCCCTC |
| MA0761.1 | ETV1 | 6.665 | 0.84161589 | GAAGGAAGTA |
| MA0761.1 | ETV1 | 5.94 | 0.8279283 | TCAGGAAGAG |
| MA0761.1 | ETV1 | 5.477 | 0.81918712 | AGAGGAAGGG |
| MA0761.1 | ETV1 | 5.067 | 0.81144656 | GAAGGATGTG |
| MA0761.1 | ETV1 | 4.847 | 0.80729308 | AAGGGAAGTT |
| MA0722.1 | VAX1 | 6.22 | 0.85945793 | CTCATGAA |
| MA0722.1 | VAX1 | 5.993 | 0.8530141 | TTCATGAG |
| MA0722.1 | VAX1 | 5.676 | 0.84401544 | CTCATTGA |
| MA0722.1 | VAX1 | 6.194 | 0.85871987 | TCAATGAG |
| MA0722.1 | VAX1 | 5.696 | 0.84458318 | CTAAAGAC |
| MA0722.1 | VAX1 | 4.258 | 0.80376278 | CACATGAC |
| MA0882.1 | DLX6 | 4.104 | 0.82019633 | TCAATGAG |
| MA0882.1 | DLX6 | 4.159 | 0.82162245 | CCCATTTC |

**Predicted putative sites in mouse SBE6.2**

| Model ID | Model name | Score | Relative score | predicted site sequence |
| --- | --- | --- | --- | --- |
| MA0882.1 | DLX6 | 4.778 | 0.83767285 | ACAATAAA |
| MA0722.1 | VAX1 | 4.603 | 0.81355627 | TTTATTGT |
| MA0747.1 | SP8 | 7.915 | 0.82915635 | TCCACCCCTACA |
| MA0722.1 | VAX1 | 4.873 | 0.82122074 | CAAATGGC |
| MA0722.1 | VAX1 | 4.775 | 0.81843882 | TTCATTTC |
| MA0882.1 | DLX6 | 3.662 | 0.80873546 | CATATTAT |
| MA0722.1 | VAX1 | 4.144 | 0.80052667 | TAAATCAC |
| MA0747.1 | SP8 | 9.031 | 0.84989642 | GTCACACCCCCA |
| MA0722.1 | VAX1 | 5.718 | 0.84520769 | CTTATGAC |

**Predicted putative sites in human SBE6.1**

| Model ID | Model name | Score | Relative score | predicted site sequence |
| --- | --- | --- | --- | --- |
| MA0761.1 | ETV1 | 5.396 | 0.81765789 | AGCGTATGTG |
| MA0761.1 | ETV1 | 5.299 | 0.81582659 | GAAGGAAATA |
| MA0882.1 | DLX6 | 6.97 | 0.89451043 | CCAATTTT |
| MA0722.1 | VAX1 | 4.727 | 0.81707625 | CCAATTTT |
| MA0747.1 | SP8 | 7.728 | 0.82568109 | CCCAGCCCCACC |
| MA0722.1 | VAX1 | 4.376 | 0.80711244 | TTCAATAC |
| MA0747.1 | SP8 | 6.661 | 0.80585165 | GACATCCCCCGT |
| MA0761.1 | ETV1 | 6.509 | 0.8386707 | GCCGGACACA |
| MA0761.1 | ETV1 | 6.995 | 0.8478461 | TGCGGAAGGG |
| MA0747.1 | SP8 | 9.489 | 0.85840802 | ACAACGCCCTCC |
| MA0722.1 | VAX1 | 4.936 | 0.82300912 | CAAATAAC |
| MA0882.1 | DLX6 | 3.671 | 0.80896883 | GCAAATAA |
| MA0761.1 | ETV1 | 8.063 | 0.86800933 | AACGGAAGGT |
| MA0761.1 | ETV1 | 4.485 | 0.80045873 | GACGGAAAAC |
| MA0747.1 | SP8 | 6.4 | 0.80100116 | GCTCCTCCTCCT |
| MA0722.1 | VAX1 | 5.21 | 0.83078713 | CTCATGGC |
| MA0722.1 | VAX1 | 6.426 | 0.86530564 | CAAATGAT |
| MA0747.1 | SP8 | 6.635 | 0.80536846 | ACAAAGCCCGCT |
| MA0631.1 | Six3 | 12.164 | 0.83658066 | AAAAGGGGATCATAAGC |
| MA0722.1 | VAX1 | 5.308 | 0.83356905 | CTTATGAT |

**Predicted putative sites in human SBE6.2**

| Model ID | Model name | Score | Relative score | predicted site sequence |
| --- | --- | --- | --- | --- |
| MA0747.1 | SP8 | 7.953 | 0.82986255 | ACCCCACCCAGA |
| MA0747.1 | SP8 | 7.145 | 0.81484645 | CCCACCCCACCC |
| MA0747.1 | SP8 | 8.476 | 0.83958214 | GCCCACCCCACC |
| MA0747.1 | SP8 | 6.641 | 0.80547997 | GCCACCTCTCCT |
| MA0882.1 | DLX6 | 6.926 | 0.89336953 | GCAATTTC |
| MA0722.1 | VAX1 | 4.386 | 0.80739631 | CCCATGAT |
| MA0882.1 | DLX6 | 3.763 | 0.81135435 | GACATTAT |
| MA0882.1 | DLX6 | 5.218 | 0.84908185 | CCAATAAT |
| MA0722.1 | VAX1 | 5.064 | 0.82664264 | CCAATAAT |
| MA0722.1 | VAX1 | 5 | 0.82482588 | CCAATGGT |
| MA0722.1 | VAX1 | 4.873 | 0.82122074 | CAAATGGC |
| MA0882.1 | DLX6 | 6.534 | 0.88320515 | GCAATTTT |
| MA0882.1 | DLX6 | 5.186 | 0.84825211 | ACAATGAA |
| MA0722.1 | VAX1 | 6.058 | 0.85485925 | TTCATTGT |
| MA0722.1 | VAX1 | 4.537 | 0.81168273 | CTCAATAC |
| MA0882.1 | DLX6 | 3.514 | 0.80489789 | CTCATCAC |
| MA0722.1 | VAX1 | 4.642 | 0.81466336 | CTCATCAC |
| MA0747.1 | SP8 | 8.445 | 0.83900602 | CCCCAGCCCACA |
| MA0761.1 | ETV1 | 4.574 | 0.802139 | AACGGCAGCA |
| MA0747.1 | SP8 | 7.937 | 0.82956521 | CCCACACCCTCA |
| MA0747.1 | SP8 | 6.723 | 0.80700388 | AACCCTCCCCAA |
| MA0747.1 | SP8 | 6.402 | 0.80103832 | ACCAACCCTCCC |
| MA0761.1 | ETV1 | 6.107 | 0.83108117 | CCCGGATGAC |
| MA0761.1 | ETV1 | 9.018 | 0.88603919 | TGCGGAAGTC |
