# Supplementary material for: SBE6: a novel long-range enhancer involved in driving sonic hedgehog expression in neural progenitor cells
Source: Open Biol. 2016 Nov 16;6(11):160197. doi: 10.1098/rsob.160197 (PMC5133441; doi:10.1098/rsob.160197)
Supplement: Supplementary Table 3 [file rsob160197supp7.docx]

### Supplementary Table 3. Cis-Regulatory Element (CRE) driven transgene expression sites in F1 zebrafish embryos

| Transgene | Reporter | Number of stable transgenic lines analysed | Sites of reporter expression | Tissue-specific activity of the CRE observed in 100% of transgenic lines analysed |
| --- | --- | --- | --- | --- |
| Shh-SBE6.1 | eGFP | 4 | **Forebrain**  **(4/4; 100%)**  Pectoral fin (1/4; 25%)  Heart (1/4; 25%)  Retina (1/4; 25%) | Forebrain |
| Shh-SBE6.2 | mCherry | 4 | Forebrain (1/4; 25%)  Otic vesicle (1/4; 25%)  Olfactory placode (1/4; 25%)  Neural tube (1/4; 25%) | None |
| Shh-SBE6.1-negative ctrl | mCherry | 2 | Faint ubiquitous signal | None |
| Shh-SBE6.2-negative ctrl | eGFP | 3 | Notocord (1/3; 33%)  Heart (1/3; 33%)  Ubiquitous (1/3; 33%) | None |

### The Table shows the sites of transgene expression from Cis-Regulatory Element (CRE) driven reporters in F1 zebrafish (*Danio rerio*) embryos obtained from multiple independent stable transgenic F0 lines. Cis-Regulatory elements were derived from the murine genome and the reporters were either GFP or mCherry as described in Bhatia et al., 2015 [27]. The negative controls were sequences downstream (3') of *Shh*, which have no suspected regulatory activity and which are the same genomic distance from the *Shh* promoter as SBE6.1 and SBE6.2 are upstream (5').

|  |  |  |  |  |
| --- | --- | --- | --- | --- |
|  |  |  |  |  |
|  |  |  |  |  |
|  |  |  |  |  |
|  |  |  |  |  |
|  |  |  |  |  |
|  |  |  |  |  |
|  |  |  |  |  |
|  |  |  |  |  |
|  |  |  |  |  |
