# Supplementary material for: SBE6: a novel long-range enhancer involved in driving sonic hedgehog expression in neural progenitor cells
Source: Open Biol. 2016 Nov 16;6(11):160197. doi: 10.1098/rsob.160197 (PMC5133441; doi:10.1098/rsob.160197)
Supplement: Supplementary Table 4 [file rsob160197supp8.docx]

### Supplementary Table 4. Primers used for gRNAs for CRISPR/Cas9 targeting of SBE6.1 and SBE6.2. PAM sites are on the external sides of the sequences.

| SBE6.1 Downstream Fw | CACCGgtcacattctccgtcattcag |
| --- | --- |
| SBE6.1 Downstream Rv | AAACctgaatgacggagaatgtgacC |
| SBE6.1 Upstream Fw | CACCGgtaggccagattacttgcaag |
| SBE6.1 Upstream Rv | AAACcttgcaagtaatctggcctacC |
| SBE6.2 Downstream Fw | CACCGgtctgtttgtagagaccttac |
| SBE6.2 Downstream Rv | AAACgtaaggtctctacaaacagacC |
| SBE6.2 Upstream Fw | CACCGgttagacactccagttttgtc |
| SBE6.2 Upstream Rv | AAACgacaaaactggagtgtctaacC |
